# Monthly Summary

**Published:** 1895-06

**Authors:** 


### Monthly Summary.

RATIONAL THERAPEUTICS OF CHOLERA INFANTUM.—BY GUSTAVUS BLECH, M. D., St. Louis.—No strict rules can be given for the treatment of disease. It is for this reason that so many physicians say we do not treat a disease, but we treat an individual. True enough, we treat the individual, but what we have most of all to consider is the disease. The individual will dictate us alterations and modifications in our treatment.

A general plan of treatment may be outlined, however, and I will try to do so in regard to one of the most fatal diseases of babyhood—cholera infantum. There is a certain philosophy in therapeutics which I would frame in the three following rules: First, remove if possible the disturbing causes; second, treat symptoms which *per se* are liable to endanger the life of the patient; and third, sustain vitality.

As said before; the therapeutics, which is based upon the ætiology and pathology of a given case is the only one to be employed.

Now, the ætiology of cholera infantum is not so obscure as asserted by a good many authors. Whether or not of microbic origin, one thing is sure—it is due to a chemical decomposition of food, causing an inflammatory condition of the digestive and alimentary canal.

Clinical experience, furthermore, shows that this disease is of a grave character, producing death in a large proportion. Heat *per se* is not the immediate cause of this disease, but it influences its course considerably. Therefore, gastric or intestinal disturbances in summer demand a closer attention than those which occur during the colder season. Cholera infantum is a disease met even in the palaces of the rich, although not so often as in the tenement houses of the poor, which fact proves again that bad air, filth and lack of ventilation are also of a predisposing influence, as well as an obstacle to a quick cure. The mortality in the tenement houses is larger than that of the richer parts.

If we consider the aforesaid, we shall first of all, as regards the treatment of this disease, have to restrict diet.

As soon as called to a case of cholera infantum, prohibit for the first day any food whatever. Mothers have no right to nurse the little patient either. Strict instructions must be given in that direction, because the timid mothers are often inclined to quiet the crying babies by putting them to the breast.

Remedies are of very little value. Beginning with calomel, salol, and all the newer antiseptics, finishing with subnitrate of bismuth—they have all proved a failure, for none of them work quickly enough.

The treatment as outlined by Dr. Elmer Lee, of Chicago, in his cases of typhoid fever, proved a success in my hands during last summer, and under this treatment I have lost only one patient out of twenty-three, while the monuments of my skill exercised during the year 1893 are decorating the cemeteries of the State of Connecticut

So far as I knew, the best antiseptic (which has also a strong tendency to reduce local inflammation) was peroxide of hydrogen (medicinal) until hydrozone was nsed by me. Hydrozone being twice as strong as Marchand's peroxide of hydrogen, (for economical reasons) the latter drug is preferred by me. This remedy can be administered internally as well as externally.

I add a tablespoonful of hydrozone to a pint of water for washing out the stomach. The vomiting ceases after the first washing as a rule. If necessary, this procedure can be repeated. If the vital power of the little patient is not too low it can produce no harm. But in every case, no matter how far advanced, I do not omit an irrigation of the bowels, for which purpose I use a soft rubber catheter attached to a common bulb syringe. The catheter is introduced as high in the colon as possible. It is unnecessary to say that the water must first be sterilized. I do not agree with Dr. Lee in using hot soap water. On the contrary, I use cold water, and add to each quart about two ounces of hydrozone. The improvement after the first or second irrigation is marked. If necessary, these irrigations can be repeated every two hours.

Among other remedies there are only two to be employed, morphine and strychnine. Both ought to be administered hypodermically. Their indication is too well known and they are about all we need. No antipyretics should be given. If the fever is very high and if the irrigation of the bowels does not redue it, the whole body should be washed with alcohol.

The diet for the next twenty-four hours should be very light indeed. Sweet, strong Russian tea is all I allow.

Each individual case will teach us when food can be allowed again.

Since the adoption of this mode of treatment I have met with the most remarkable success, and no honest practitioner should refuse it a trial.

---

To be successful in filling teeth with gold, be sure to have your undercuts filled. To do this, small pieces of gold must be used to start the filling, large pieces do not fill them as solid as the foundation of a filling should be.—*Traveling Dentist.*

---

HORACE WELLS ANNIVERSARY PROCEEDINGS.—The chairman of the executive committee of the Horace Wells 30th Anniversary Celebration, announces that the papers read by Profs. Fillebrowne and Garrettson at the meeting, and the speeches delivered at the banquet have been prepared for publication in the proposed Souvenir Volume, and will be issued upon the receipt of a sufficient number of subscriptions to cover the expense. Price $1.50.

The undersigned will receive subscriptions, receipt for same, and deliver the book upon completion.

J. D. THOMAS, Chairman.

THE FIRST PERMANENT MOLAR.—Dr. Palmer, of Janesville, Wis., in a paper before the Wisconsin State Dental Society, has some very pertinent thoughts on the first permanent molar. Although this tooth, from its size, perfection of outline and position in the jaw, is the most important, yet it is the worst treated of any tooth in the mouth. Owing to the extraordinary strain on the system of the child during the growth of this tooth, it is generally inferior to the others in structure, and parents hold more wrong ideas of it than of all the other teeth combined. The extracting of this tooth is a fruitful source of arrested development of the alveolar ridge and subsequent irregularities. The tipping forward of the second molar which follows extraction of the first, causes a very serious alteration in the articulation, and the anterior teeth suffer as a consequence, being pushed out of position, changing the whole expression of the face. Again, the removal of these teeth will cause the eruption of the teeth anterior to them to be retarded, as the occlusion of the jaws should come on the first molars. Dentists should impress upon their patients at every opportunity the importance of attention to these teeth.—*Dental Record.*

---

FOR VERY SOFT TEETH.—By T. B. Welch.—To harden the dentine under a cement filling, rub on a little tannin made into a thin paste with oil of cloves. Fill with oxiphosphate. The softened dentine soon becomes "tanned" and a permanent covering is made for the pulp.—*Items of Interest.*

---

A LIQUID WHICH WILL MIX ALMOST ANY CEMENT FILLING.—Metaphosphoric acid glacial in sticks, 1 oz., dissolved in aqua dist. 3 ozs. Cork the bottle firmly, and let it stand for twenty-four hours. Then put the liquid in an evaporating dish, and let it boil over a medium gas-jet for about five or six minutes; fill in a well-corked bottle, and when cooled down it is ready for use. This liquid will always remain clear.—*Dental Weekly.*

WATER CURE.—Water can cure more people by far than is generally supposed. Water is able to create a centrifugal power and aids in the elimination from the body of effete material. No surgeons can ever make such an opening as is made by nature when supported by water. In this way, I often think that though such small operations as are done by dentists on structures of low vitality are indispensable, yet the therapy of all oral diseases of inflammatory character may be materially aided by water treatment. For instance, if we wish to get rid of effete material in a root canal, it would be a good method to introduce something, such as linen fibers soaked with water, which creates a centrifugal power and helps to remove it. It would then be cleaned. A root canal can not become clean by introducing drugs and leaving behind effete material. Sterilization is not so perfect in the removal of such material as the centrifugal power created by the proper application of water.—Dr. H. Block in *Items of Interest.*

---

WHITE FILLINGS.—We hear that New York dentists have almost ceased to put gold in the mouths of ladies o fashion. Unless the filling is quite out of sight, most of them prefer to have the best white filling used, and to then visit the dentist often to have it renewed as it wears away. A fortune awaits the person who will find a white filling that does not wear away.—*Dental Weekly.*

---

CINNAMON DRINK FOR FEVER PATIENTS.—Cinnamon tea is recommended by a Southern physician as a valuable drink in fever-affected districts. It possesses an especial virtue against typhoid fever, and essence of cinnamon is said to be one of the best of disinfectants to use in the sick room of a typhoid patient.—*Items of Interest.*

ENGLISH DENTAL EXAMINATIONS.—Mr. Thomas Gaddes, of Harrogate, has been writing to the *Lancet* on the dentist's education, and urges the Royal College of Surgeons of England to lose no time in effecting improvements in the nature and scope of its dental examinations. He says:—The examinations of candidates for the L. D. S. of the Royal College of Surgeons of England has long been a grievance with dental teachers. Beyond the introduction of the 'practical' portion in 1879 no other improvement of any moment has taken place in the examination since 1872, when written papers were given to the *viva voce*. All the other licensing corporations—Edinburgh, Glasgow, and Dublin—have their primary and final examinations at two periods of the dental student's career. But in this matter the Royal College of Surgeons of England remains stationary, apparently fixed, notwithstanding the utterances of teachers and others, many times repeated from 1879, urging that the examination in the general subjects of the curriculum—at all events, anatomy and physiology—be taken at a different time from that of certain of the special subjects—dental surgery, &c. Not only would such a division of the examination reduce the tendency to 'cram' which the existing single examination in all the subjects fosters, but the onus of preparing the candidates in anatomy and physiology would rest upon the teachers of those sciences at the general medical schools, and not, as heretofore and at present, upon the tutors of the dental school—a preposterous anomaly which ought not to exist."—*Dental Weekly.*

---

TO REMOVE REMNANTS OF PUTRESCENT TISSUE IN ROOT-CANALS.—By C. N. Peirce.—Force trichloracetic acid into the canals. It destroys all tissues and purifies in a few moments' time.—*Items of Interest.*

---

RUBBER dissolved in chloroform is one of the most useful articles in the laboratory for repairing cases.—*Dominion Dental Journal.*

SOME time since a merchant came into my office bringing an orange some one had bitten into.

"Doctor," said he, "can you tell who bit into that orange?"

"Yes," we said; "it was a young man for whom I am making a part set of teeth."

"Can you swear to it?"

"Yes; here is the articulation I took of his mouth only yesterday."

"What was his name?"

"Joseph Fairbanks."

The articulation fitted the bite into the orange precisely. The left lateral was turned; the right cuspid was prominent, and the right central I was to restore, was absent.

While the family were at church he had broken into the house and stolen money and other valuables. The orange was one of three left on the center table, too sour to eat.

He was convicted and sent to prison.—Editor *Items.*

---

TO BLEACH A TOOTH.—By Dr. Meeker.—Apply caustic pyrozone by means of bibulous paper twisted on a gold probe. Move it around in the cavity, throwing on a current of air to hasten evaporation.—*Items of Interest.*

---

SHEEP WITH GOLDEN TEETH.—On the teeth of sheep and other ruminants, a metallic shining covering is observed. In Germany the occurrence is rare, but in southern countries, like Sardina, Sicily, Creta, Greece, Syria, Mesopotamia, Persia, Egypt, etc., it is more frequent. There the covering is considered as real gold, and gave rise to the tradition that the sheep owe their "golden teeth" to a wonderful plant, 'gold cabbage."

This plant shines at night and possesses charming powers. It is known among the Greek shepherds as lampidonia, *i. e.*, the illuminating. There is a tradition that wise men know how to obtain gold from the gold cabbage,

The most curious thing about the matter is that the shepherds asked the learned men to find that gold cabbage for them. As to the gold shining covering of the sheep's teeth, different examinations have imposed the conclusion that it is a sediment from the saliva, and therefore a metal shining tartar.

---

ROOT-CANAL FILLING.—Make a saturated solution of aristol in chloroform. Having cleaned and dried the canals, with a dropper fill with the aristol solution and quickly introduce a gutta-percha point in each canal. Simple, efficient and inexpensive.—*Georgia Practitioner.*

---

FACIAL PARLAYSIS.—The conclusions drawn from the observation of eighty cases of facial paralysis by Dr. Rudolph Hatschek are detailed in the *Jahrbuch fuer Psychiatrie und Neurologie.* A short summary of the paper appears in the *Neurologisches Centralblatt.* Among the eighty cases were ten in which more than one attack had occurred. Sometimes one side, sometimes both sides were affected, and more than once there were third attacks. There seemed to be no tendency for one sex to suffer more than the other, and there was no preference for one side of the face over the other. As a rule the latter attack was more severe, but not invariably so. Two of the cases occurred in patients the subjects of diabetes; one occurred in a patient at the commencement of the secondary manifestations of syphilis. One case of double facial paralysis occurred in a boy eight years of age after mumps; in another patient, a lad of seventeen years of age, an attack of right-sided facial paralysis succeeded to sore throat with pyrexia. It is also mentioned as an interesting fact that during an epidemic illness (apparently influenza) at Brest facial paralysis was one of the recognized symptoms.—*London Lancet.*

